# 5′-tRF-19-Q1Q89PJZ Suppresses the Proliferation and Metastasis of Pancreatic Cancer Cells via Regulating Hexokinase 1-Mediated Glycolysis

**DOI:** 10.3390/biom13101513

**Published:** 2023-10-12

**Authors:** Wenpeng Cao, Zhirui Zeng, Shan Lei

**Affiliations:** 1 Department of Anatomy, School of Basic Medicine, Guizhou Medical University, Guiyang 550025, China; 2 Department of Physiology, School of Basic Medicine, Guizhou Medical University, Guiyang 550025, China; zengzhirui@gmc.edu.cn

**Keywords:** pancreatic cancer, 5′-tRF-19-Q1Q89PJZ, hexokinase 1, glycolysis, metastasis

## Abstract

tRNA-derived small RNAs (tDRs) are dysregulated in several diseases, including pancreatic cancer (PC). However, only a limited number of tDRs involved in PC progression are known. Herein, a novel tDR, 5′-tRF-19-Q1Q89PJZ (tRF-19-Q1Q89PJZ), was verified in PC plasma using RNA and Sanger sequencing. tRF-19-Q1Q89PJZ was downregulated in PC tissues and plasma, which was related to advanced clinical characteristics and poor prognosis. tRF-19-Q1Q89PJZ overexpression inhibited the malignant activity of PC cells in vitro, while tRF-19-Q1Q89PJZ inhibition produced an opposite effect. The differentially expressed genes induced by tRF-19-Q1Q89PJZ overexpression were enriched in “pathways in cancer” and “glycolysis”. Mechanistically, tRF-19-Q1Q89PJZ directly sponged hexokinase 1 (HK1) mRNA and inhibited its expression, thereby suppressing glycolysis in PC cells. HK1 restoration relieved the inhibitory effect of tRF-19-Q1Q89PJZ on glycolysis in PC cells and on their proliferation and mobility in vitro. tRF-19-Q1Q89PJZ upregulation inhibited PC cell proliferation and metastasis in vivo and suppressed HK1 expression in tumor tissues. Furthermore, tRF-19-Q1Q89PJZ expression was attenuated under hypoxia. Collectively, these findings indicate that tRF-19-Q1Q89PJZ suppresses the malignant activity of PC cells by regulating HK1-mediated glycolysis. Thus, tRF-19-Q1Q89PJZ may serve as a key target for PC therapy.

## 1. Introduction

Pancreatic cancer (PC) is one of the leading causes of cancer-related mortality worldwide [1]. In recent years, scholarly research has been primarily dedicated to the identification of biomarkers that may play a significant role in facilitating early detection, thereby reducing the incidence of illness and death [2]. The timely diagnosis of pancreatic cancer holds the potential to enhance patient prognosis through the implementation of curative surgical interventions. Consequently, directing endeavors toward the identification of individuals harboring high-risk or precursor lesions emerges as a crucial aspect in augmenting survival rates [3]. Hence, the imperative lies in the development of diagnostic tools that enable the prompt and precise identification of pancreatic cancer, as this stands as a pivotal measure in mitigating mortality rates and bolstering overall survival outcomes.

Recently, reprogramming of energy metabolism has been recognized as a distinct feature of tumors, including PC [4]. To ensure prompt proliferation and provide a material basis for DNA synthesis, PC cells generate energy through the rapid glycolysis approach, even in the presence of abundant oxygen, which is also called the “Warburg effect”. As such, several enzymes involved in glycolysis are elevated in PC tissues, which is related to the poor prognosis of patients with this malignancy [5,6]. Further, elevated glycolysis promotes PC cell proliferation and metastasis through activating a series of pathways, highlighting that targeting glycolysis is a powerful strategy to treat PC [7,8].

Small RNAs (tDRs) derived from tRNAs are usually 14–50 nucleotides long [9,10]. Depending on the location of biogenesis, tDRs can generally be divided into tRNA-derived small RNAs (tsRNAs), tRNA halves (tiRNAs), and tRNA-derived fragments (tRFs) [11]. In addition, tRFs can be further divided into three subgroups: 5′-tRF, 3′-tRF, and inter-tRF (i-tRF) [12]. They are functionally diverse and associated with the regulation of gene expression, RNA processing, ribosome biogenesis, and long terminal repeat retrotransposons [13]. Several human diseases are associated with dysregulation of tDRs, including cancer, virus infection, metabolic disorders, and neurodegenerative diseases [14]. Specifically, tsRNA-26576 was more highly expressed in breast cancer tissues compared with that in the adjacent tissues, while tsRNA-26576 knockdown reduced the proliferation and metastasis of breast cancer cells [15]. Moreover, 5′tiRNA-His-GTG levels were elevated in colorectal cancer, and 5′tiRNA-His-GTG overexpression was related to tumor size [16]. Furthermore, tsRNA-MetCAT-37 and tsRNA-ValTAC-41 levels were elevated in PC tissues and showed a remarkable diagnostic value in distinguishing tumorous from the adjacent non-tumorous tissues [14]. There is a growing body of evidence that supports the existence of abundant tDRs in various human cell lines, tissues, and extracellular body fluids. Recent studies have shown that tDRs can differentiate between pre- and post-seizure patients [17]. Notably, a significant increase in tDRs has been observed in the plasma exosomes of liver cancer patients, with four specific tDRs showing promise as potential diagnostic biomarkers [18]. However, only a limited number of tDRs that regulate the malignant properties of PC are known.

To this end, in the present study, we identified the involvement of tDRs in the development of PC and explored their biological functions and the underlying molecular mechanisms in PC cells. Here, 5′-tRF-19-Q1Q89PJZ (tRF-19-Q1Q89PJZ) was downregulated in PC tissues, and it inhibited the proliferation and metastasis of PC cells by regulating hexokinase 1 (HK1)-mediated glycolysis. Thus, tRF-19-Q1Q89PJZ may serve as a novel and potent target for PC therapy.

## 2. Materials and Methods

### 2.1. Ethical Statement

Samples were collected from 20 patients diagnosed with PC and 20 healthy individuals with no history of basic or chronic diseases. The samples were obtained from the Hepatological Surgery Department at the Affiliated Hospital of Guizhou Medical University in Guiyang, China, using ethylene diamine tetraacetic acid (EDTA) anticoagulation tubes. The blood samples were immediately centrifuged at 4 °C, 1200 relative centrifugal force (RCF), for 10 min, and the plasma was collected. Subsequently, the plasma was stored at −80 °C and used for the experiments. The plasma samples were collected from patients at the time of diagnosis, prior to any surgical, chemotherapeutic, radiative, or other forms of treatment. To assess the relationship between the expression of tRF-19-Q1Q89PJZ in both PC tissues and plasma from the same patients, a total of 80 paired fresh tumor tissues were obtained from patients who underwent curative resection at the Affiliated Hospital of Guizhou Medical University. The samples were obtained during surgical procedures, without any prior administration of chemotherapy or radiotherapy. The specimens were subsequently preserved at a temperature of −80 °C until they were utilized. Details of all samples are provided in Table S3.

### 2.2. Small RNA Library Preparation and Sequencing

Total RNA was extracted from the PC and HC plasma using the TRIzol reagent (Beyotime, Suzhou, China). RNA integrity and quantity were examined prior to library construction. The rtStar™ tRF&tiRNA Pretreatment Kit and First-Strand cDNA Synthesis Kit (Aksomics Inc., Shanghai, China) were used to remove mRNA epigenetic modifications for promoting efficient cDNA reverse transcription (RT). Next, 3′- and 5′-small RNA adapters were ligated to cDNA using the GoScript™ Reverse Transcription Mix (Promega, Madison, WI, USA). RNA fragments of 135–160 bp were amplified and used for library construction. Finally, the library was analyzed using the Agilent 2100 Bioanalyzer (Agilent, Santa Clara, CA, USA). Trimmed reads were aligned with the reference sequences of mature tRNA and pre-tRNA. Finally, the R (version 4.0.2, Armonk, NY, USA) package edgeR was used to analyze differentially expressed tDRs, with a threshold of *p* < 0.05 and |LogFC| ≥ 1.

### 2.3. qRT-PCR

Total RNA was extracted from the PC tissues and cells using the TRIzol reagent. Before RT, the rtStar™ tRF&tiRNA Pretreatment Kit and First-Strand cDNA Synthesis Kit were used to remove mRNA epigenetic modifications. tDRs were quantified by qRT-PCR using the Bulge Loop miRNA Stater Kit (RIBOBIO, Guangzhou, China), following the manufacturer’s protocol, and reverse transcribed to cDNA with stem-loop RT primers specific to 5′-half-32-PNR8YP9LON4V3, 5′-half-33-LQ947673FEWSD3, 3′-tRF-22-WE8S68L52, 5′-tRF-19-Q1Q89PJZ, and 5′-tRF-35-LQ947673FEWS3V. The RT flow for tDRs was as follows: 60 min at 42 °C, 5 min at 70 °C, and 10 min at 4 °C. SYBR Green (Yeasen, Shanghai, China) was used for qRT-PCR. The U6 small nuclear 6 pseudogene (RNU6B) was used for tsRNA template normalization. For RT of HK1, homeobox D3 (HOXD3), RAB GTPase binding effector protein 2 (RABEP2), zinc finger protein 74 (ZNF74), and β-actin (ACTB), the PrimeScript RT reagent Kit (Takara, Japan) was used, and the conditions were as follows: 15 min at 37 °C, 5 s at 85 °C, and 10 min at 4 °C. ACTB was used as the reference gene for HK1, HOXD3, RABEP2, and ZNF74. The amplification and fluorescence quantification conditions were as follows: 30 s at 95 °C, 40 cycles of 5 s at 95 °C and 30 s at 60 °C, and 10 s at 95 °C. Primers for tDRs were obtained from RIBOBIO (https://www.ribobio.com/ (accessed on 12 April 2022)). For patent protection, the primer sequences used for tDRs cannot be made public but can be consulted from the connecting manufacturer. The other primer sequences used were the following:

HOXD3 Forward: 5′-AGAGTCTCGACAGAACTCCAAG-3′,
Reverse: 5′-GTTCCGTGAGATTCAGCAGGT-3′.
RABEP2 Forward: 5′-TGCCTGCACCATGAGGTAAAG-3′,
Reverse: 5′-CGTCACGATCTCGATCCGC-3′.
HK1 Forward: 5′-GCTCTCCGATGAAACTCTCATAG-3′,
Reverse: 5′-GGACCTTACGAATGTTGGCAA-3′.
ZNF74 Forward: 5′-AGAACTACCAGAACCTTCTTGCC-3′,
Reverse: 5′-CGCCTCGTTCCAGATGAGAG-3′.β-actin Forward: 5′-GGAGCGAGATCCCTCCAAAAT-3′,Reverse: 5′-GGCTGTTGTCATACTTCTCATGG-3′.

### 2.4. Cell Culture and Lentivirus Infection

The PC cell lines MIA-PaCa2, PANC-1, CFPAC-1, Capcan-2, SW1990, BxPC-3, AsPC-1, and human pancreatic epithelium immortalized cells (HPDE) were obtained from Procell (Wuhan, China). All cells were cultured in Dulbecco’s modified Eagle’s medium (DMEM; Gibco, Grand Island, NY, USA) supplemented with 10% fetal bovine serum (Gibco, Grand Island, NY, USA). The cells were cultured at 37 °C in an incubator with 5% CO_2_. tRF-19-Q1Q89PJZ-knockdown lentivirus and the control (anti-NC) were obtained from Biomedicine Biotech (Chongqing, China). The tRF-19-Q1Q89PJZ sequence and the control (anti-NC) were cloned into pSuper-retro-puro to construct pSuper-retro-tRF-19-Q1Q89PJZ-RNAi. Lentiviruses overexpressing HK1, tRF-19-Q1Q89PJZ, and the control (tRF-NC) were constructed by subcloning the PCR-amplified full-length human HK1 and tRF-19-Q1Q89PJZ cDNA into the pMSCV retroviral plasmid. Polybrene (Biomedicine Biotech, Chongqing, China) was used for lentivirus infection. Briefly, stable PC cells were treated with 0.5 μg·mL^−1^ of puromycin for 12 days, followed by lentivirus infection.

### 2.5. Cell Proliferation Detection

The Cell Counting Kit-8 (CCK-8) and 5-ethynyl-2 (EDU) assays were performed to measure cell proliferation. For the CCK-8 assay, PC cells were seeded at a density of 3000 cells/well in a 96-well plate. Following cell adhesion for 0, 24, 48, 72, and 96 h, 100 μL of fresh DMEM containing 1/10 CCK-8 reagent (Servicebio, Wuhan, China) was added to each well. After culturing for 2 h, the absorbance (OD) of each well was measured at 450 nm. The EDU assay was performed using the BeyoClick™ EdU-488 Cell Proliferation Kit (Beyotime, Shanghai, China) according to the manufacturer’s protocol. The EDU-positive cell index was determined by calculating the ratio of EDU-positive cells to total cells using ImageJ software (version 1.8.0, MD, USA).

### 2.6. Wound Healing Assay

PC cells were seeded in 6-well plates and cultured until the degree of convergence was >95%. Then, a 200 μL tip was used to vertically scratch the monolayer cell to generate a wound. After washing the floating cell mass, FBS-free medium was added. The wound conditions at 0 h and 24 h were recorded under an inverted optical microscope (Axiovert 200; Carl Zeiss, Oberkochen, Germany). The migration rate of PC cells was referenced to the wound healing area. ImageJ software (version 1.8.0, MD, USA) was used to analyze the wound healing percentage.

### 2.7. Transwell Assay

FBS-free DMEM was used to resuspend the PC cells at a density of 1 × 10^6^ cells·mL^−1^, and 200 μL of cell suspension was added to the upper chamber (Invitrogen, Carlsbad, CA, USA) pre-coated with Matrigel (Invitrogen, Carlsbad, CA, USA). Then, 600 μL of DMEM containing 10% FBS was added to the lower chamber. To the upper chamber, 4% paraformaldehyde for 20 min and 0.5% crystal violet for 15 min were applied, in that order, followed by scrubbing. Finally, invasive cells in the upper chamber were observed using an orthotopic light microscope (Axioscope5; Carl Zeiss).

### 2.8. RNA Sequencing and Enrichment Analysis

Total RNA from PANC-1 cells overexpressing tRF-19-Q1Q89PJZ and tRF-NC cells was extracted using the TRIzol reagent. Library construction and RNA sequencing were performed at Lc-bio (Hangzhou, China). Bioanalyzer 2100 and RNA 6000 nanochip kits were used to measure the amount and purity of total RNA. High-quality RNAs with RIN > 7.0 were screened for library construction. Finally, 300 ± 50 bp cDNA was used for terminal sequencing using Illumina Novaseq. Count number was set as a unit to evaluate gene expression. The differentially expressed genes (DEGs) between PANC-1 cells overexpressing tRF-19-Q1Q89PJZ and NC cells were analyzed using the R package (R package, version 3.3.3) edgeR. The cutoff for selecting DEGs was set at *p* < 0.05 and |LogFC| ≥ 1.

### 2.9. Extracellular Acidification Rate (ECAR) and O_2_ Consumption Rate (OCR) Measurement

The XF Glycolysis Stress Test Kit (Seahorse Bioscience) and XF Cell Mito Stress Test Kit (Seahorse Bioscience) were used to determine the ECAR and OCAR of PC cells. PC cells (1 × 10^4^ cells/well) were seeded into 96-well XF cell culture microplates. Glucose (100 mM), oligomycin (10 μM), and 2-deoxyglucose (50 mM) were added, in that order, at specific time points to determine the ECAR of PC cells. Oligomycin (5 μM), FCCP (10 μM), and rotenone (10 μM) plus antimycin A (5 μM) were added to determine the OCR.

### 2.10. Detection of Glucose Uptake and Lactate and ATP Production

The Glucose Uptake Colorimetric Assay kit (Merck, NJ, USA) was used to detect glucose uptake by PC cells. PC cells were seeded in a 96-well plate with HEPES buffer, and then a 20 min glucose uptake assay was performed following the addition of 2-deoxyglucose (10 mM). A lactic acid test kit (Thermo Fisher Scientific, Waltham, MA, USA) and an ATP detection kit (Thermo Fisher Scientific, Waltham, MA, USA) were used to determine lactate and ATP production, respectively. PC cells (1 × 10^6^ cells) were homogenized in 100 μL of the corresponding buffers and evaluated using a bioluminmeter. The relative values of lactate, glucose uptake, and ATP levels were normalized to the control group (set to 1).

### 2.11. Dual-Luciferase Reporting Assay

PC cells were seeded into 6-well plates and transfected with an NC/tRF-19-Q1Q89PJZ mimic and a luciferin enzyme HK1 reporter carrying the wildtype (WT)/mutant-type (MUT) binding sites for 4–6 h. Then, the cells were cultured at 37 °C for 24 h. The Dual-Luciferase Reporter Assay System (Promega, Madison, WI, USA) was used to determine the luciferase activity in cells.

### 2.12. Western Blotting

Total protein in PC cells was extracted using radioimmunoprecipitation assay (RIPA) buffer (Solarbio, Beijing, China) containing 1:100 PMSF (Solarbio, Beijing, China). A BCA kit (Solarbio, Beijing, China) was used to measure the protein concentration. Then, proteins from each sample were separated by 10% SDS–PAGE (Solarbio, Beijing, China) and transferred onto PVDF membranes (Invitrogen, Carlsbad, CA, USA). After blocking with 5% BCA for 1 h, the membranes were incubated with primary antibodies for HOXD3 (Cat No. A15279; 1:400, Abconal, Wuhan, China), HK1 (Cat No. A0533; 1:500, Abconal, Wuhan, China), RABEP2 (Cat No. 14625-1-AP; 1:500, Proteintech, Wuhan, China), ZNF74 (Cat No. 21843-1-AP; 1:500, Proteintech, Wuhan, China), and β-actin (Cat No. AC026; 1:5000, Abconal). After washing two times with TBST and incubation with secondary antibodies, the membranes were visualized using the Gene Genius Bioimaging System (Bio-Rad, CA, USA) using the ECL reagent (Yeasen, Shanghai, China). Gene expression was normalized to β-actin expression.

### 2.13. In Vivo Experiments

The proliferation and metastasis of PC cells were studied in vivo using the subcutaneous tumorigenesis and pulmonary metastasis models, respectively. To create the subcutaneous tumorigenesis model, the right flank of mice was subcutaneously injected with 2 × 10^6^ AsPC-1 cells. On day 28, the mice were sacrificed after their health conditions were monitored daily and tumor volume was measured using calipers weekly. Tumor size was calculated every three days using the following equation: (length × width^2^)/2. Tumor tissues were extracted and subjected to immunohistochemical staining. To create the pulmonary metastasis model, 2 × 10^6^ AsPC-1 cells were injected into the caudal vein of mice. The health condition of mice was monitored daily, and when the mice developed dyspnea, the experiment was terminated, and all mice were sacrificed. The pulmonary tissues of mice were extracted and used to count the metastatic foci. The Guizhou Medical University Animal Ethics Committee approved the in vivo experiments (approval number: 2200187).

### 2.14. Immunohistochemical Stain

The tumor tissues were cut into 2 mm-thick paraffin-embedded sections, dewaxed with xylene, and rehydrated in a graded series of alcohol. The tumor sections were restored using sodium citrate at a high temperature and pressure. To prevent nonspecific binding, 5% bicinchoninic acid (BCA) and 3% H_2_O_2_ were used for 30 min. The tumor sections were incubated with primary antibodies for Ki-67 (Cat No. A16919; 1:200, Abconal), PCNA (Cat No. A12427; 1:200, Abconal), and HK1 (Cat No. A0533; 1:100, Abconal) at 4 °C overnight. After washing two times with PBS and incubating with secondary antibodies, the antigen–antibody complexes in the tumor sections were visualized using DAB staining. After washing with PBS, gene expression patterns in the tumor sections were assessed using a light orthophoto microscope (Olympus, Tokyo, Japan).

### 2.15. RNA Fluorescence In Situ Hybridization

The tRF-19-Q1Q89PJZ probe was acquired from Ribobio (Guangzhou, China). PANC-1 and AsPC-1 cells were cultured and fixed in 4% paraformaldehyde for 30 min at 37 °C. Following PBS washing and permeabilization with 0.1% Triton-X-100 for 10 min, the cells were subjected to a 2 h incubation with the probes. DAPI was employed to stain the cell nuclei for 5 min, and the resulting images were captured using a fluorescence microscope (ZEISS, Germany).

### 2.16. In Situ Hybridization

The RNA ISH Kit (BersinBi, Beijing, China) was utilized in the experiments, following the instructions provided by the manufacturer. Initially, PC cells were fixed in a 4% paraformaldehyde solution for a duration of 20 min. Subsequently, the cells were washed with distilled water, treated with pepsin (1% in 10 mM HCl), and incubated with an ISH probe of 20 nM concentration in a hybridization buffer (consisting of 100 mg/mL of dextran sulfate and 10% formamide in 2 × SSC) at a temperature of 90 °C for a duration of 3 min. The hybridization process was then carried out at a temperature of 37 °C for a duration of 18 h, followed by a washing step and an incubation with DAB for 1 h. Finally, the application of DAB to the samples facilitated the detection of the signals. The ISH images were captured using a light orthophoto microscope.

### 2.17. Nucleocytoplasmic Separation Assay

The nucleocytoplasmic separation assay was conducted to determine the subcellular localization of tRF-19-Q1Q89PJZ in PC. Following the established protocol of the PARIS™ Kit (Ambion, Austin, TX, USA), total RNA was fractionated into nuclear and cytoplasmic components. The isolated cytoplasmic and nuclear RNA fractions were subsequently utilized for qPCR analysis. β-actin and LaminB1 were employed as internal reference genes for cytoplasmic and nuclear RNA, respectively.

### 2.18. Statistical Analysis

The results are presented as mean ± standard deviation. GraphPad Prism 6.0 (San Diego, CA, USA) was used for statistical analyses. Kaplan–Meier curves were generated to compare the overall survival rates between the two groups. An unpaired *t*-test was used to determine the differences between two groups, and one-way analysis of variance, followed by Tukey’s test, was used to determine the differences among multiple groups. *p* < 0.05 was considered significant.

## 3. Results

### 3.1. Screening of Differentially Expressed tDRs in PC Tissues

**Figure 1:**
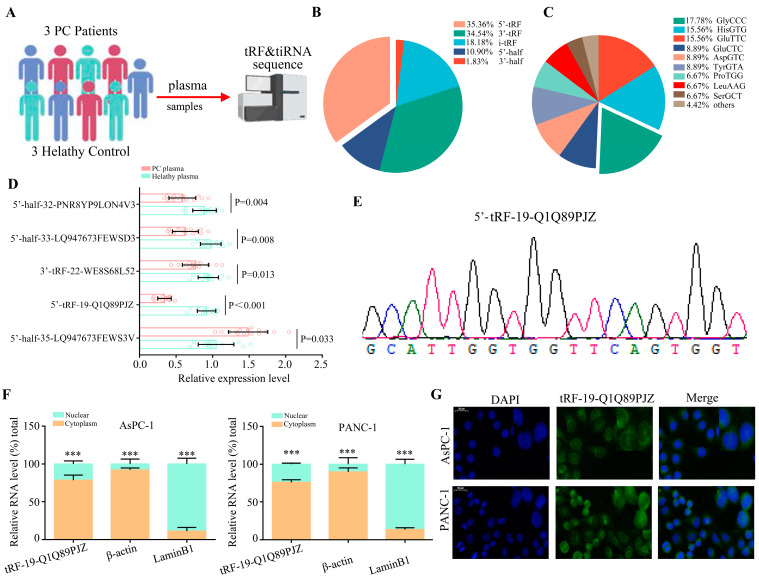
The expression profiles of tDRs in plasma of PC and HC. (**A**) Schematic presenting the experimental procedure of our study. (**B**) Percent in differential tDRs for each type in PC and HC plasma. (**C**) The expression profiles in PC and HC plasma. (**D**) Candidate tDRs’ expression as measured using qRT-PCR. (**E**) qRT-PCR product confirmed using Sanger sequencing. (**F**,**G**) Nucleocytoplasmic separation and FISH assays revealed that tRF-19-Q1Q89PJZ is mainly expressed in the cytoplasm in AsPC-1 and PANC-1 cells. *** *p* < 0.001, *n* = 3. The control group was used for comparison. Data are shown as mean ± SD.

In order to investigate the expression profiles of tDRs in plasma of PC and healthy controls (HC), small RNAs ranging from 10 to 50 bp were isolated from plasma samples of 3 PC and 3 HC subjects (Figure 1A). A total of 31 upregulated and 27 downregulated tDRs were detected in plasma of PC and HC (Figure S1A,B). Furthermore, high-throughput small RNA sequencing revealed that the abundance of differential tDRs in PC plasma decreased in the order of 5′-tRF  > 3′-tRF > i-tRF > 5′-half  > 3′-half (Figure 1B). The abundance of differential tDRs in PC plasma decreased in the order of GlyCCC > HisGTG/GluTTC > GluTTC/AspGTC/TyrGTA (Figure 1C). We further checked its expression in plasma of patients with PC (*n* = 20) and HC (*n* = 20). Our data showed that the abundance of tRF-19-Q1Q89PJZ was significantly higher in HC patients than in PC (Figure 1D). A MINTbase search revealed that it belongs to a class of 19-nt small RNAs characterized by the 5′-GCGCCGCTGGTGTAGTGGT-3′ sequence (Figure S1C). tRF-19-Q1Q89PJZ originated from the 5′-end of mature tRNA-Gly-CCC-2-2, carrying the anticodon CCC (Figure S1D). Further, Sanger sequencing was applied to determine the PCR product, and the results were consistent with the sequence information obtained from MINTbase v2.0, with perfect matching (Figure 1E). The qRT-PCR was used to determine the expression of tRF-19-Q1Q89PJZ in PC cell lines (MIA-PaCa2, PANC-1, CFPAC-1, Capcan-2, SW1990, BxPC-3, AsPC-1), and it was lower compared with that in human pancreatic epithelium immortalized cells (HPDE), where AsPC-1 and PANC-1 were the most significant (Figure S1E). Furthermore, nuclear/cytoplasmic RNA quantification and the FISH assay exhibited that tRF-19-Q1Q89PJZ transcription was prominent to the cytoplasm in AsPC-1 and PANC-1 cells (Figure 1F,G).

### 3.2. tRF-19-Q1Q89PJZ Is Downregulated in PC

**Figure 2:**
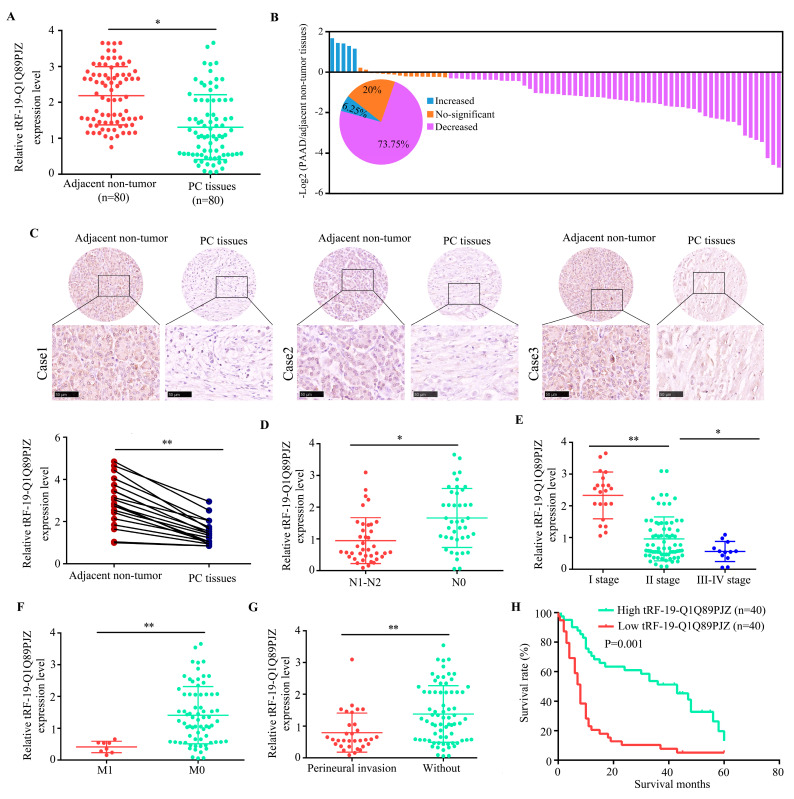
Correlation between tRF-19-Q1Q89PJZ expression and clinical characteristics of PC. (**A**) Expression level of tRF-19-Q1Q89PJZ as detected using qRT-PCR in 80 paired PC and adjacent non-tumor tissues. (**B**) Fold changes (log2) in tRF-19-Q1Q89PJZ expression in each paired sample, arranged from high to low values. (**C**) ISH analysis of the expression level of tRF-19-Q1Q89PJZ in PC and adjacent non-tumor tissue. (**D**) Correlation between tRF-19-Q1Q89PJZ expression and lymph node invasion. (**E**) Correlation between tRF-19-Q1Q89PJZ expression and clinical stage. (**F**) Correlation between tRF-19-Q1Q89PJZ expression and distant metastasis. (**G**) Correlation between tRF-19-Q1Q89PJZ expression and perineural invasion. (**H**) PC cases were divided into two groups according to the median value of tRF-19-Q1Q89PJZ expression. Overall survival was analyzed using Kaplan–Meier survival analysis with the log-rank test. * *p* < 0.05, ** *p* < 0.01, *n* = 3. The control group was used for comparison. Data are shown as mean ± SD.

We further evaluated the tRF-19-Q1Q89PJZ levels in PC tissues and observed that tRF-19-Q1Q89PJZ was downregulated in PC tissues by ISH and qRT-PCR detection (Figure 2A,C). Of the 80 paired clinical samples, 59 cases (73.75%) exhibited lower tRF-19-Q1Q89PJZ expression in tumor than in paired non-tumor tissues (Figure 2B). To further validate the effect of tRF-19-Q1Q89PJZ in PC, we examined the correlation between tRF-19-Q1Q89PJZ expression and the clinicopathological parameters of patients with PC. tRF-19-Q1Q89PJZ expression was lower in the PC tissues of patients with regional lymph node invasion (Figure 2D), advanced clinical stage (Figure 2E), metastasis (Figure 2F), and perineural invasion (Figure 2G; Table S1). Survival analysis of 80 PC patients was conducted using information available at the clinical follow-up. The results of the 5-year follow-up visits showed that 15 cases survived (survival = 0) and 65 cases died (death = 1) among the 80 cases. The 5-year overall survival rate was 18.8% (15/80) and the death rate was 81.2% (65/80). Furthermore, we used the median value of the tRF-19-Q1Q89PJZ expression level as the cutoff to classify PC tissues with high and low tRF-19-Q1Q89PJZ expression. Patients with PC exhibiting lower tRF-19-Q1Q89PJZ expression showed shorter survival times (Figure 2H).

### 3.3. tRF-19-Q1Q89PJZ Suppresses PC Cell Proliferation and Mobility In Vitro

**Figure 3:**
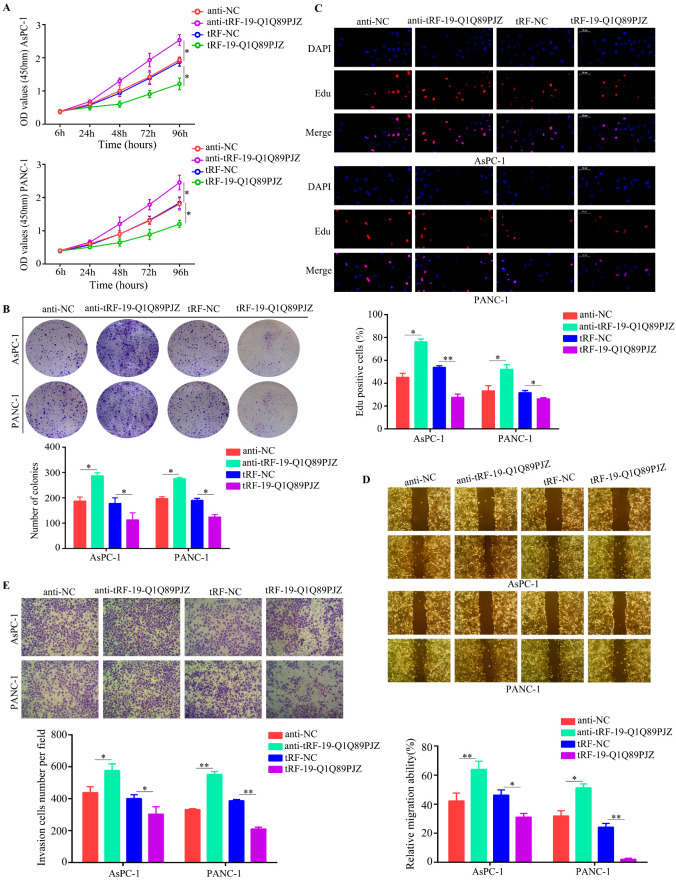
tRF-19-Q1Q89PJZ inhibits the proliferation and metastasis of PC cells. (**A**) CCK-8 assay comparing the proliferation of PANC-1 and AsPC-1 cells in the anti-NC (negative control), the anti-tRF-19-Q1Q89PJZ, tRF-NC, and tRF-19-Q1Q89PJZ groups. (**B**) Colony formation assay of PANC-1 and AsPC-1 cells in the anti-NC, anti-tRF-19-Q1Q89PJZ, tRF-NC, and tRF-19-Q1Q89PJZ groups. (**C**) EDU assay of the proliferation of PANC-1 and AsPC-1 cells in the anti-NC, anti-tRF-19-Q1Q89PJZ, tRF-NC, and tRF-19-Q1Q89PJZ groups. (**D**) Wound healing assay of the migration ability of PANC-1 and AsPC-1 cells in the anti-NC, anti-tRF-19-Q1Q89PJZ, tRF-NC, and tRF-19-Q1Q89PJZ groups. (**E**) Transwell assay of the migration and invasion ability of PANC-1 and AsPC-1 cells in the anti-NC, anti-tRF-19-Q1Q89PJZ, tRF-NC, and tRF-19-Q1Q89PJZ groups. * *p* < 0.05, ** *p* < 0.01, *n* = 3. The control group was used for comparison. Data are shown as mean ± SD.

Next, we used a targeted tRF-19-Q1Q89PJZ inhibitor/mimic to inhibit/augment tRF-19-Q1Q89PJZ expression in PC cells (Figure S2A,B). The CCK-8 (Figure 3A) and colony formation assays (Figure 3B) showed that tRF-19-Q1Q89PJZ downregulation significantly increased the viability and colony-forming ability of AsPC-1 and PANC-1 cells in vitro, whereas tRF-19-Q1Q89PJZ overexpression produced opposite effects. Higher EDU-positive rates were observed in AsPC-1 and PANC-1 cells with tRF-19-Q1Q89PJZ inhibition, whereas lower EDU-positive rates were observed in PC cells with tRF-19-Q1Q89PJZ overexpression (Figure 3C). Moreover, tRF-19-Q1Q89PJZ inhibition remarkably promoted the migration and invasion ability of AsSPC-1 and PANC-1 cells in vitro, while tRF-19-Q1Q89PJZ overexpression inhibited this effect (Figure 3D,E).

### 3.4. tRF-19-Q1Q89PJZ Suppresses Glycolysis in PC Cells

**Figure 4:**
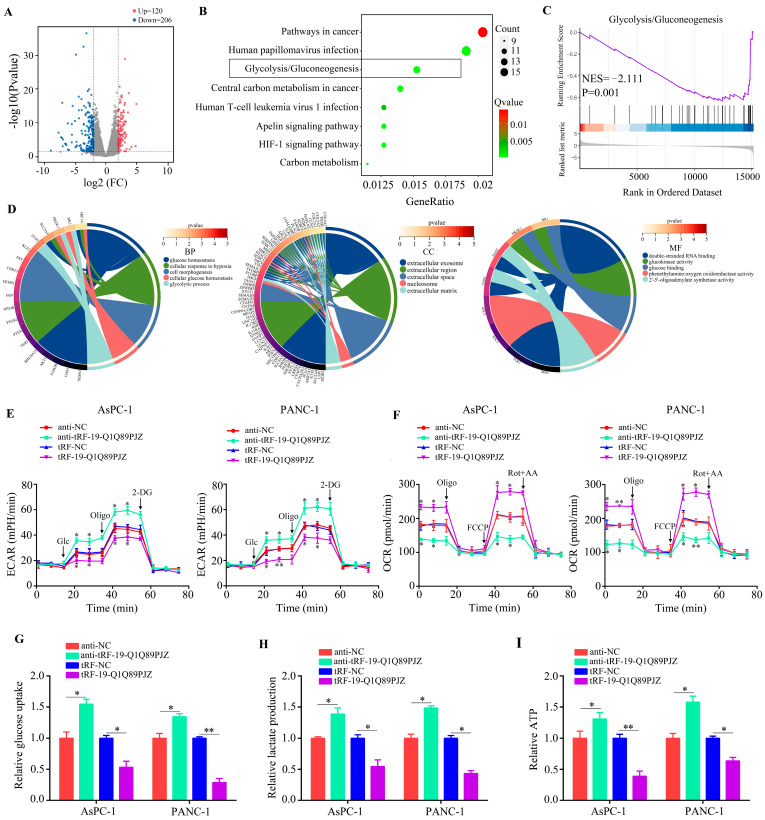
tRF-19-Q1Q89PJZ inhibits glycolysis in PC cells. (**A**) Volcano plot showing mRNA levels between PANC-1 cells transfected with tRF-NC and tRF-19-Q1Q89PJZ. (**B**) KEGG analysis was performed to determine the pathways in which the differentially expressed genes were enriched. (**C**) Gene enrichment plots showing a series of genes enriched in glycolysis/gluconeogenesis. (**D**) GO analysis was performed to determine the pathways of differentially expressed genes enriched in glycolysis/gluconeogenesis. (**E**,**F**) Seahorse Bioscience XFp measured the ECAR and OCR of PANC-1 and AsPC-1 cells in the anti-NC, anti-tRF-19-Q1Q89PJZ, tRF-NC, and tRF-19-Q1Q89PJZ groups. (**G**–**I**) Glucose uptake, lactate production, and ATP synthesis of PANC-1 and AsPC-1 cells in the anti-NC, anti-tRF-19-Q1Q89PJZ, tRF-NC, and tRF-19-Q1Q89PJZ groups. * *p* < 0.05, ** *p* < 0.01, *n* = 3. The control group was used for comparison. Data are shown as mean ± SD.

RNA sequencing was used to detect the internal regulatory network of tRF-19-Q1Q89PJZ in PC cells. Through performing RNA sequencing and DEG analysis, 120 upregulated and 206 downregulated genes were identified between PANC-1 cells overexpressing tRF-19-Q1Q89PJZ and tRF-NC (negative control) (Figure 4A). Through KEGG analysis, these 326 DEGs were found to enrich in cancer, human papillomavirus infection, glycolysis/gluconeogenesis, central carbon metabolism in cancer, the HIF-1 signaling pathway, the apelin signaling pathway, human T-cell leukemia virus 1 infection, and carbon metabolism (Figure 4B). Gene set enrichment analysis (GSEA) revealed that tRF-19-Q1Q89PJZ negatively regulates glycolysis (Figure 4C). Furthermore through GO analysis, MF terms associated with differentially expressed genes were enriched in “double-stranded RNA binding”, “glucokinase activity”, “glucose binding”, “phenethylamine: oxygen oxidoreductase activity”, and “oligoadenylate synthetase activity”. BP terms were enriched in “glucose homeostasis”, “cellular response to hypoxia”, “cell morphogenesis”, “cellular glucose homeostasis”, and “glycolytic process”. CC terms were enriched in the “extracellular exosome”, “extracellular region”, “extracellular space”, “nucleosome”, and “extracellular matrix” (Figure 4D).

Activation of the glycolysis/gluconeogenesis signaling pathway is a key mediator of the malignant behavior of PC cells; therefore, we focused on this pathway. In ECAR analysis, both basal and maximal ECAR were decreased in the tRF-19-Q1Q89PJZ-overexpressing group than in the tRF-19-Q1Q89PJZ-knockdown group (Figure 4E). Similarly, OCR analysis indicated that tRF-19-Q1Q89PJZ overexpression increased oxygen consumption in PC cells, whereas tRF-19-Q1Q89PJZ knockdown reduced oxygen consumption (Figure 4F). In addition, glucose uptake and lactate and ATP production were remarkably reduced in PC cells overexpressing tRF-19-Q1Q89PJZ. Meanwhile, tRF-19-Q1Q89PJZ knockdown significantly increased glucose uptake, lactate production, and ATP synthesis (Figure 4G–I). Collectively, these results indicate that tRF-19-Q1Q89PJZ inhibits glycolysis in PC cells.

### 3.5. tRF-19-Q1Q89PJZ Directly Targets HK1, Whereas HK1 Overexpression Rescues the Suppressive Effect of tRF-19-Q1Q89PJZ on Glycolysis

**Figure 5:**
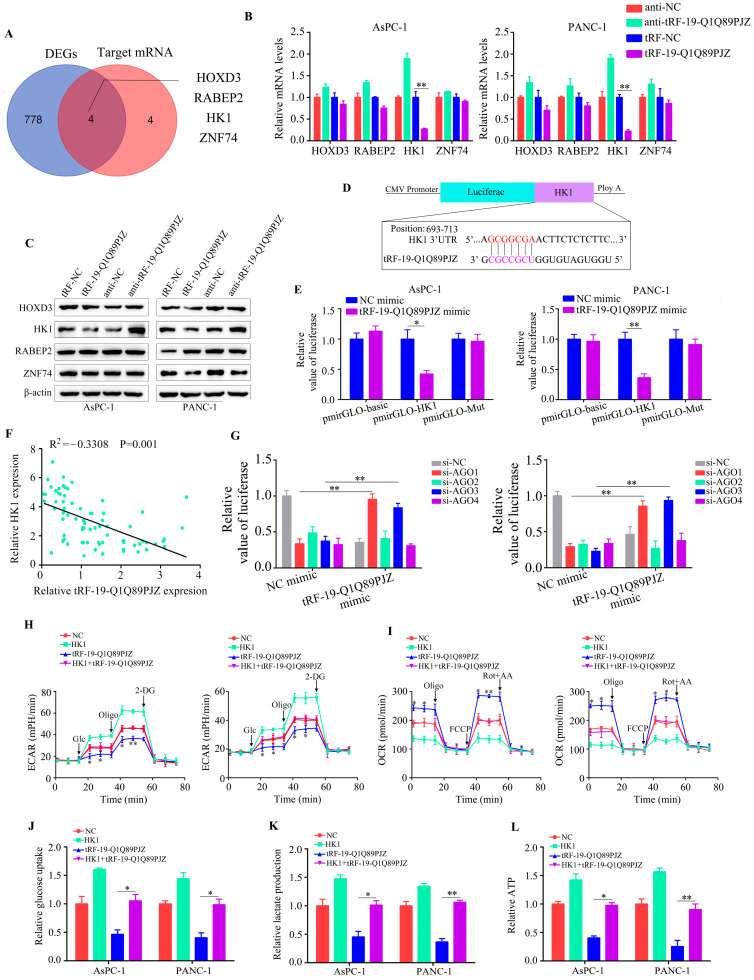
HK1 is a target of tRF-19-Q1Q89PJZ in PC. (**A**) Venn diagram showing overlapping of the identified and predicted target mRNAs of tRF-19-Q1Q89PJZ. (**B**) qRT-PCR showing the transcript levels of HOXD3, HK1, RABEP2, and ZNF74 in PC cells transfected with anti-tRF-19-Q1Q89PJZ or tRF-19-Q1Q89PJZ, or their negative controls. (**C**) Western blotting showing the protein levels of HOXD3, HK1, RABEP2, and ZNF74 in PC cells transfected with anti-tRF-19-Q1Q89PJZ or tRF-19-Q1Q89PJZ, or their negative controls. (**D**) Schematic diagram of the predicted interaction position between tRF-19-Q1Q89PJZ and seed regions within the 3′-UTR and mutation region of HK1. (**E**) Luciferase activity of pmirGLO-HK1 was significantly decreased by the tRF-19-Q1Q89PJZ mimic in PC cells. (**F**) Spearman rank correlation analysis, showing a negative correlation between tRF-19-Q1Q89PJZ and HK1. (**G**) PC cells were co-transfected with a plasmid expressing the HK1 3′-UTR, tRF-19-Q1Q89PJZ mimic, and siRNAs targeting AGO1, AGO2, AGO3, and AGO4. After 48 h, the transfected cells were collected using the luciferase assay. (**H**,**I**) Seahorse Bioscience XFp measured the ECAR and OCR of PANC-1 and AsPC-1 cells in the NC, HK1, tRF-19-Q1Q89PJZ, and HK1+tRF-19-Q1Q89PJZ groups. (**J**–**L**) Glucose uptake, lactate production, and ATP synthesis of PANC-1 and AsPC-1 cells in the NC, HK1, tRF-19-Q1Q89PJZ, and HK1+tRF-19-Q1Q89PJZ groups. * *p* < 0.05, ** *p* < 0.01, *n* = 3. The control group was used for comparison. Data are shown as mean ± SD.

The tRF–target mRNA interaction commonly induces the degradation or translational repression of target mRNAs. First, bioinformatic algorithms were applied, which revealed eight potential target genes of tRF-19-Q1Q89PJZ. Second, mRNA-Seq was used to detect changes in the related genes after overexpression of tRF-19-Q1Q89PJZ. There were 206 genes that were downregulated and 120 genes that were upregulated, based on |LogFC| ≥ 2 and an adjusted *p* < 0.05 (Figure 4A; Table S2). After overlapping the mRNA-Seq-identified DEGs (n = 326) and the potential target genes of tRF-19-Q1Q89PJZ predicted by bioinformatics, four intersection genes: HOXD3, RABEP2, HK1, and ZNF74, were identified (Figure 5A). Among these, HK1 mRNA and protein levels were reduced or increased the most significantly in PC cells with tRF-19-Q1Q89PJZ overexpression or inhibition (Figure 5B,C). Therefore, we constructed HK1 dual-reporter fluorescence plasmids with wildtype and mutant binding sites (Figure 5D) and transfected them into PC cells. tRF-19-Q1Q89PJZ obviously reduced the fluorescence intensity in PC cells transfected with HK1 dual-reporter fluorescence plasmids carrying wildtype binding sites, but not in those carrying mutant binding sites (Figure 5E). Interestingly, according to data from TCGA and our research cohort, HK1 was overexpressed in PC tissues (Figure S3A,B), and HK1 overexpression was positively associated with poor prognosis (Figure S3C,D). Moreover, tRF-19-Q1Q89PJZ expression was negatively associated with HK1 expression (Figure 5F). Argonaute (AGO) family proteins are involved in tDRs’ production. Therefore, to determine whether AGO proteins affected tRF-19-Q1Q89PJZ expression, AGO1, AGO2, AGO3, and AGO4 were knocked down, and changes in luciferase expression were observed. tRF-19-Q1Q89PJZ-mediated luciferase expression was remitted by AGO1 and AGO3 inhibition (Figure 5G) but not by AGO2 and AGO4 inhibition. Therefore, AGO1 and AGO3 facilitate the tRF-19-Q1Q89PJZ-mediated regulation of HK1 expression, implying that HK1 serves as the target gene of tRF-19-Q1Q89PJZ. To determine whether HK1 is involved in the inhibitory effect of tRF-19-Q1Q89PJZ on glycolysis, we restored HK1 expression in tRF-19-Q1Q89PJZ-overexpressing PC cells. HK1 restoration in tRF-19-Q1Q89PJZ-overexpressing PC cells increased ECAR (Figure 5H) and decreased OCR (Figure 5I). Similarly, elevated HK1 expression in tRF-19-Q1Q89PJZ-overexpressing PC cells increased cell glucose uptake (Figure 5J), lactate production (Figure 5K), and ATP synthesis (Figure 5L).

### 3.6. Restoration of HK1 Reverses the Inhibitory Effect of tRF-19-Q1Q89PJZ on PC Cell Proliferation and Mobility

**Figure 6:**
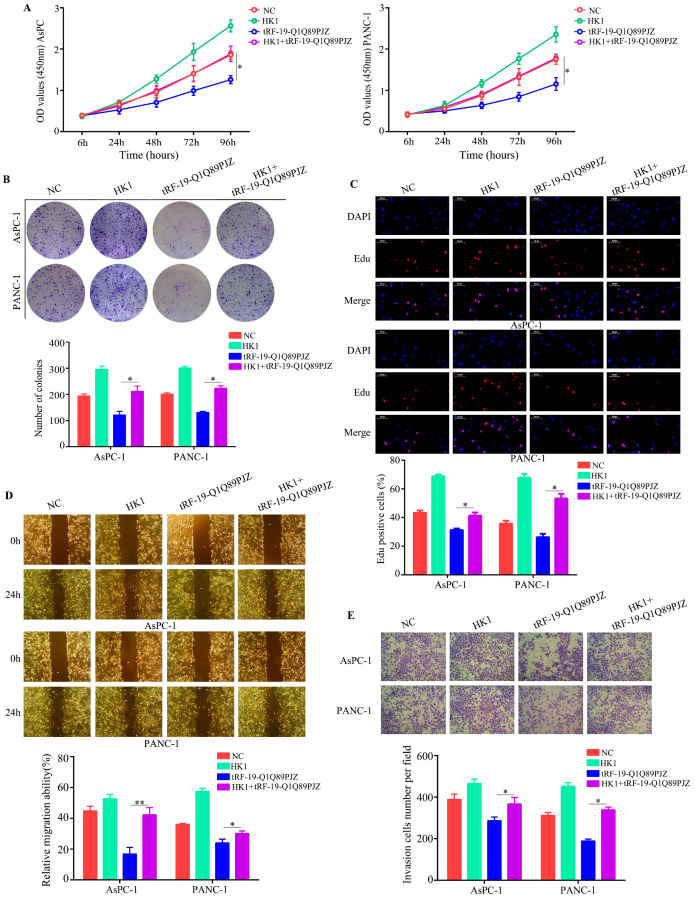
Restoration of HK1 reverses the inhibitory effects of tRF-19-Q1Q89PJZ. Cells were divided into four groups and subjected to different treatments: negative-control lentiviruses transfection (NC), transfection of tRF-19-Q1Q89PJZ lentiviruses (tRF-19-Q1Q89PJZ) alone, transfection of HK1 plasmid (HK1) alone, and transfection of tRF-19-Q1Q89PJZ lentiviruses and HK1 plasmid (HK1+ tRF-19-Q1Q89PJZ). (**A**) CCK-8 assays were used to detect the proliferation ability of each group. (**B**) Colony formation assays were used to determine the proliferation ability of cells in each group. (**C**) EDU assays were used to determine the proliferation ability of cells in each group. (**D**) Wound healing assays were used to determine the migration ability of cells in each group. (**E**) Transwell assays were used to determine the invasion ability of cells in each group. * *p* < 0.05, ** *p* < 0.01, *n* = 3. The control group was used for comparison. Data are shown as mean ± SD.

To determine whether HK1 is involved in the biological functions of tRF-19-Q1Q89PJZ, we restored its expression in PC cells overexpressing tRF-19-Q1Q89PJZ. HK1 overexpression evidently remitted the inhibitory effects of tRF-19-Q1Q89PJZ on cell viability and colony-forming ability (Figure 6A,B). Similarly, EDU assays indicated that HK1 restoration elevated the EDU-positive rate in PC cells overexpressing tRF-19-Q1Q89PJZ (Figure 6C). Further, wound healing and transwell assays verified that HK1 restoration reversed the inhibitory effects of tRF-19-Q1Q89PJZ on PC cell migration and invasion (Figure 6D,E).

### 3.7. tRF-19-Q1Q89PJZ Suppresses the Proliferation and Metastasis of PC Cells In Vivo

**Figure 7:**
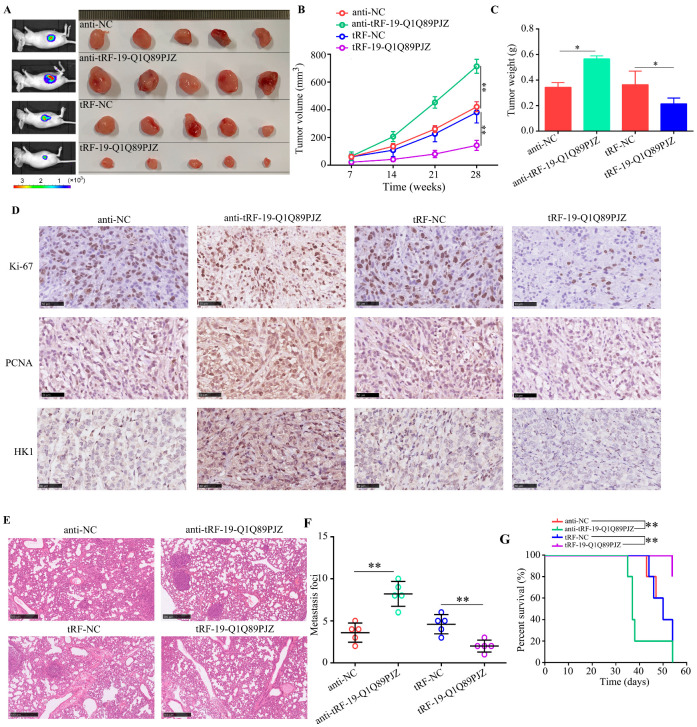
tRF-19-Q1Q89PJZ inhibits the proliferation and metastasis of PC cells in vitro. (**A**) Typical image of nude mouse tumors (*n* = 5). Subcutaneous tumor (**B**) volume and (**C**) weight. (**D**) Typical IHC staining images showing Ki-67, PCNA, and HK1 expression levels in transplanted tumors under different experimental conditions. (**E**,**F**) Typical IHC staining of HE images showing metastatic loci in the pulmonary tissues. (**G**) Kaplan–Meier survival curves for each experimental group. * *p* < 0.05, ** *p* < 0.01, *n* = 3. The control group was used for comparison. Data are shown as mean ± SD.

To determine the in vivo functions of tRF-19-Q1Q89PJZ, subcutaneous xenograft and pulmonary metastasis models were constructed. Tumor tissues originating from AsPC-1 cells with tRF-19-Q1Q89PJZ knockdown grew faster in vivo and were heavier; meanwhile, tRF-19-Q1Q89PJZ overexpression decreased the proliferation rate of tumors originating from AsPC-1 cells and their weight (Figure 7A–C). Furthermore, tumor tissues originating from AsPC-1 cells with tRF-19-Q1Q89PJZ knockdown showed higher HK1, Ki-67, and PCNA expression levels than those originating from control cells, whereas HK1, Ki-67, and PCNA expression levels were lower in tumor tissues derived from AsPC-1 cells overexpressing tRF-19-Q1Q89PJZ (Figure 7D). The results of pulmonary metastasis model indicated that AsPC-1 cells with tRF-19-Q1Q89PJZ knockdown exhibited higher metastatic ability than control cells in vivo, and fewer metastatic foci were detected in the pulmonary tissues of mice injected with tRF-19-Q1Q89PJZ-overexpressing AsPC-1 cells (Figure 7E,F). Survival analyses showed that the tRF-19-Q1Q89PJZ-overexpressing group had the longest survival time, while the knockdown group had shorter survival times (Figure 7G).

### 3.8. tRF-19-Q1Q89PJZ Is Downregulated in a Hypoxic Microenvironment

**Figure 8:**
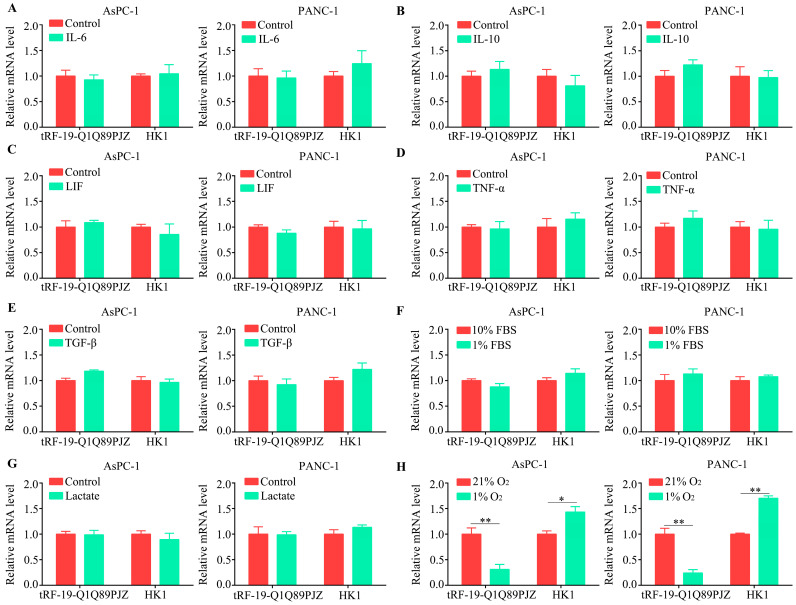
tRF-19-Q1Q89PJZ is downregulated in a hypoxic microenvironment. (**A**–**E**) Transcript levels of tRF-19-Q1Q89PJZ in PC cells cultured with inflammatory cytokines (i.e., IL-6, IL-10, LIF, TNF-α, and TGF-β dissolved in 0.5% BSA) were determined. (**F**) PC cells were cultured with 10% or 1% FBS, and the transcript levels of tRF-19-Q1Q89PJZ were detected using qRT-PCR. (**G**) PC cells were cultured in environments with or without 10 mM of lactate, and the transcript levels of tRF-19-Q1Q89PJZ were detected using qRT-PCR. (**H**) PC cells were cultured in 21% or 1% O_2_ environments, and the transcript levels of tRF-19-Q1Q89PJZ were detected using qRT-PCR. * *p* < 0.05, ** *p* < 0.01, *n* = 3. The control group was used for comparison. Data are shown as mean ± SD.

The tRF expression is driven by the tumor microenvironment. Therefore, tRF-19-Q1Q89PJZ and HK1 mRNA levels were measured in mimetic hypoxic, nutrient-depleted, inflammatory, and acidotic microenvironments using qRT-PCR. IL-6 (Figure 8A), IL-10 (Figure 8B), LIF (Figure 8C), TNF-α (Figure 8D), and TGF-β (Figure 8E) were used to simulate inflammatory microenvironments, but no significant changes were noted in the tRF-19-Q1Q89PJZ and HK1 transcript levels in these microenvironments. Next, 1% FBS and 10 mM of lactate were used to simulate nutrient-depleted (Figure 8F) and acidotic microenvironments (Figure 8G), respectively, and no significant changes were noted in the tRF-19-Q1Q89PJZ and HK1 transcript levels in these microenvironments. Finally, 1% O_2_ was used to simulate a hypoxic environment, and while the tRF-19-Q1Q89PJZ transcript levels decreased under hypoxia, the HK1 transcript levels increased (Figure 8H).

## 4. Discussion

PC, a common digestive malignancy, exhibits rapid growth and metastasis at the early stages [19]. tRNA-derived small RNAs (tDRs) were initially identified in the urinalysis of cancer patients in 1977 [20]. Subsequent literature has increasingly indicated that tDRs are not mere byproducts of random tRNA degradation, but rather possess specific biological functions in disease processes. The pivotal role of tRNA-derived fragments (tRFs) in pathology was first suggested in 2009, when Yeung et al. observed upregulation of the 3’-tRF in response to HIV infection [21]. Further research has confirmed that tDRs play a regulatory role in gene expression and contribute to various biological processes, such as development, differentiation, inflammation, and tumor oncogenesis [22]. Furthermore, a growing body of evidence has demonstrated that transfer RNA-derived fragments possess the ability to be actively secreted and persist as enduring entities within the peripheral blood. This is primarily attributed to their safeguarding against degradation by RNase activity through encapsulation within microvesicles, such as exosomes, or by forming resilient macromolecular complexes [23]. Considering the diverse composition and enduring nature of tDRs in the bloodstream, they emerge as promising contenders for cancer detection and diagnosis. Therefore, the identification of key mediators involved in PC progression may contribute to effective therapy. tDRs were initially identified as degradation products of tRNAs and regarded as “waste” in cells [24]. However, recent studies have indicated that tDRs are involved in a series of biological processes, such as cell proliferation, migration, and differentiation [25,26]. In addition, tDRs’ dysregulation has been observed in a series of cancers, including PC [27,28]. Herein, we demonstrated that tRF-19-Q1Q89PJZ, a novel tDR, was downregulated in PC plasma and tissues, whereas tRF-19-Q1Q89PJZ overexpression reduced PC cell proliferation and metastasis through inhibiting KH1-mediated glycolysis.

Specific tDRs involved in PC have previously been explored. For instance, Sui et al. [29] demonstrated that tRF-Leu-AAG was upregulated in PC tissues and was related to poor prognosis; meanwhile, knockdown of its protein significantly reduced PC cell proliferation and mobility. In another study, Cao et al. [30] indicated that tRF-19-PNR8YPJZ was upregulated in PC tissues and showed diagnostic value in distinguishing patients with PC. tRF-19-Q1Q89PJZ was downregulated in CCA tissues, affected the progress of the tumor, and has the potential to be a CCA diagnostic biomarker and therapeutic target [31]. However, tDRs involved in the malignant biological properties of PC remain largely unknown. In this context, the present study provides the first evidence that tRF-19-Q1Q89PJZ, a novel 5′-tRF, is downregulated in PC tissues, particularly of patients at the advanced stages, the M1 stage, the N1–N2 stage, and with perineural invasion. Similarly, low tRF-19-Q1Q89PJZ expression was related to poor outcomes. In contrast, tRF-19-Q1Q89PJZ overexpression reduced PC cell proliferation and metastasis, whereas tRF-19-Q1Q89PJZ knockdown induced opposite effects. Therefore, we provide the first evidence that tRF-19-Q1Q89PJZ may be a suppressive factor for PC.

Furthermore, we applied RNA-Seq to uncover the regulatory network of tRF-19-Q1Q89PJZ in PC cells and found that the DEGs induced by tRF-19-Q1Q89PJZ were enriched in glycolysis. Through GSEA, we found that tRF-19-Q1Q89PJZ is negatively associated with glycolysis. Cancer cells, including PC cells, prefer glycolysis—also known as the Warburg effect. Compared with that of aerobic glucose metabolism, the ATP production rate of glycolysis is 100 times faster. Similarly, glycolysis produces various pentose phosphates for DNA replication, thereby promoting cell proliferation. Therefore, glycolysis is a key mediator of PC progression. Enhanced glycolysis by the lncRNA HIF1A-AS1 has been shown to promote the AKT pathway and enhance gemcitabine resistance in PC cells [32]. Likewise, glycolysis induced by the oncogene dynamin 1 has been shown to promote the epithelial–mesenchymal transition and metastasis of PC cells. In the present study, we found that tRF-19-Q1Q89PJZ inhibition increased ECAR, glucose uptake, lactate production, and ATP synthesis, but reduced OCR, whereas tRF-19-Q1Q89PJZ overexpression induced the opposite effects. Therefore, tRF-19-Q1Q89PJZ regulates glycolysis in PC cells.

HK1 is a key enzyme involved in glycolysis, and it catalyzes the conversion of hexose to hexose 6-phosphate [33]. HK1 is overexpressed in a series of cancers, including PC [34,35], and elevated HK1 levels are associated with poor prognosis in PC [34]. Moreover, high HK1 expression enhanced gemcitabine resistance in PC cells [36]. HK1 is directly regulated by KRAS4A and promotes PC cell metastasis [37]. Herein, we showed, for the first time, that HK1 is one of the key target genes of tRF-19-Q1Q89PJZ, as tRF-19-Q1Q89PJZ overexpression suppressed HK1 expression. Moreover, tRF-19-Q1Q89PJZ expression was negatively associated with HK1 expression in PC tissues, and HK1 restoration reversed the suppressive effects of tRF-19-Q1Q89PJZ on the glycolysis, proliferation, and metastasis of PC cells.

Dicers are double-stranded RNA (dsRNA) endoribonucleases that induce post-transcriptional gene silencing [38], and dicers can produce tDRs by processing them [39]. The tumor microenvironment has been proven to regulate tDRs’ production through affecting the expression and activity of dicers [40,41]. Thus, to identify the driving factors for tRF-19-Q1Q89PJZ downregulation in PC cells, we exposed PC cells to a series of simulated microenvironments and confirmed that hypoxia is a key factor reducing tRF-19-Q1Q89PJZ expression in PC. In conclusion, tRF-19-Q1Q89PJZ suppresses the malignant activity of PC via regulating HK1-mediated glycolysis.

## 5. Conclusions

In this study, we have successfully identified the participation of tDRs in the progression of pancreatic cancer (PC) and have investigated their biological functions and the underlying molecular mechanisms in PC cells. We have observed a downregulation of 5′-tRF-19-Q1Q89PJZ in PC tissues. Furthermore, we have found that tRF-19-Q1Q89PJZ exerts inhibitory effects on the proliferation and metastasis of PC cells through its regulation of hexokinase 1 (HK1)-mediated glycolysis. These findings suggest that tRF-19-Q1Q89PJZ holds promise as a novel and potent therapeutic target for the treatment of PC.

## Data Availability

Not applicable.

## Supplementary Materials

The following supporting information can be downloaded at: https://www.mdpi.com/article/10.3390/biom13101513/s1.

## Abbreviations

tDRs	tRNA-derived small RNAs
PC	Pancreatic cancer
HK1	Hexokinase 1
tiRNAs	tRNA halves
tRFs	tRNA-derived fragments
CCK-8	Cell Counting Kit-8
FISH	RNA fluorescence in situ hybridization
ISH	in situ hybridization
ECAR	Extracellular acidification rate
