# Supplementary material for: 5′-tRF-19-Q1Q89PJZ Suppresses the Proliferation and Metastasis of Pancreatic Cancer Cells via Regulating Hexokinase 1-Mediated Glycolysis
Source: Biomolecules. 2023 Oct 12;13(10):1513. doi: 10.3390/biom13101513 (PMC10605356; doi:10.3390/biom13101513)
Supplement: Supplementary file 1 [file biomolecules-13-01513-s001.zip › biomolecules-2523299-supplementary.pdf]

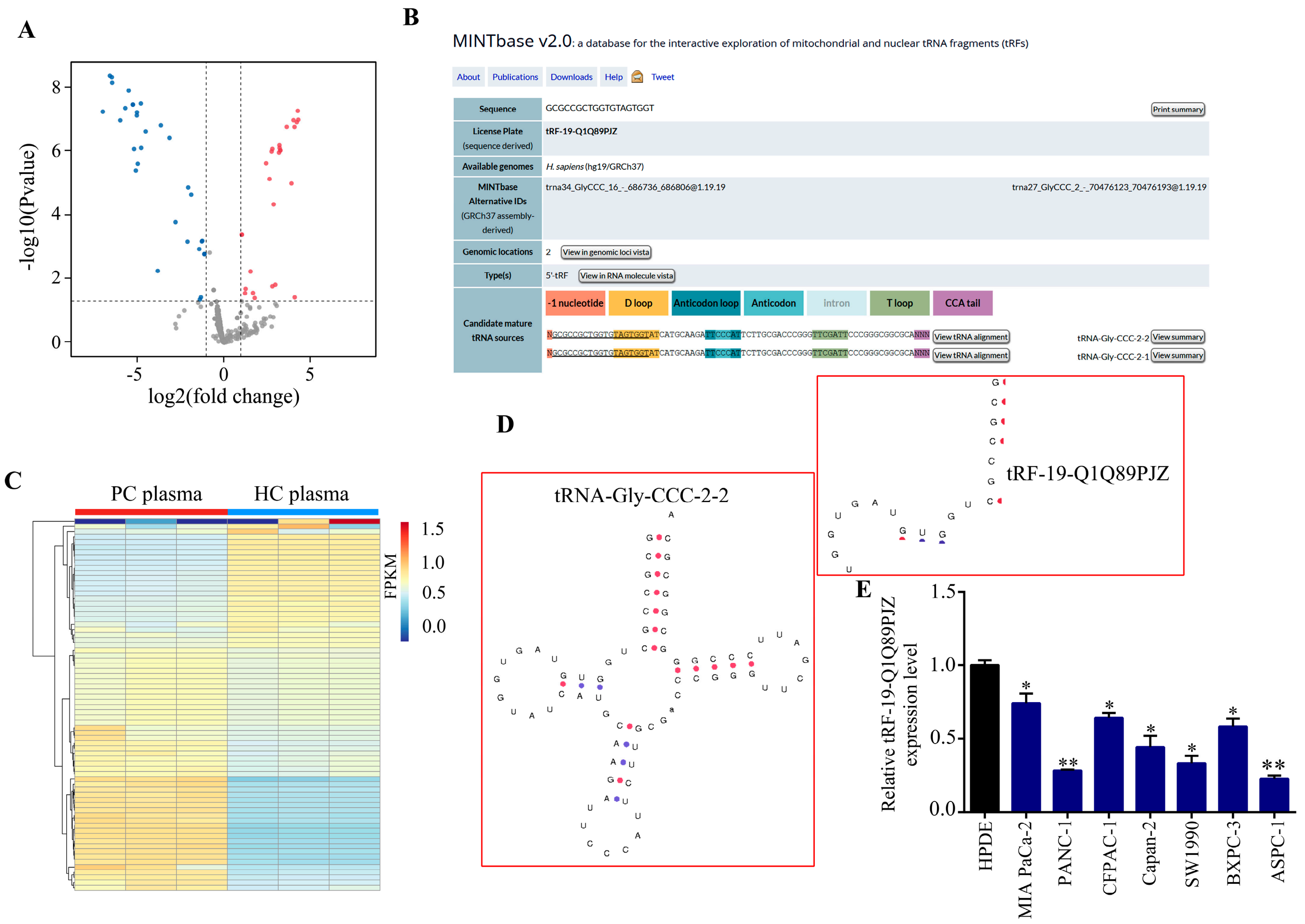

Figure S1. tRF-19-Q1Q89PJZ is a type of tRNA-derived fragment. (**A**) Volcano plot of PC versus HC. Differentially expressed tDRs are indicated in red and green. (**B**) Heat map of tDRs' expression data obtained from the cancerous and adjacent normal tissues. (**C**,**D**) tRF-19-Q1Q89PJZ is derived from the 5′-end of a 17-nt mature tRNA-Gly-CCC-2-2. (**D**) tRF-19-Q1Q89PJZ structure. (**E**) qRT-PCR analysis of relative expression levels of tRF-19-Q1Q89PJZ in pancreatic cancer cell lines and HPDE cells.

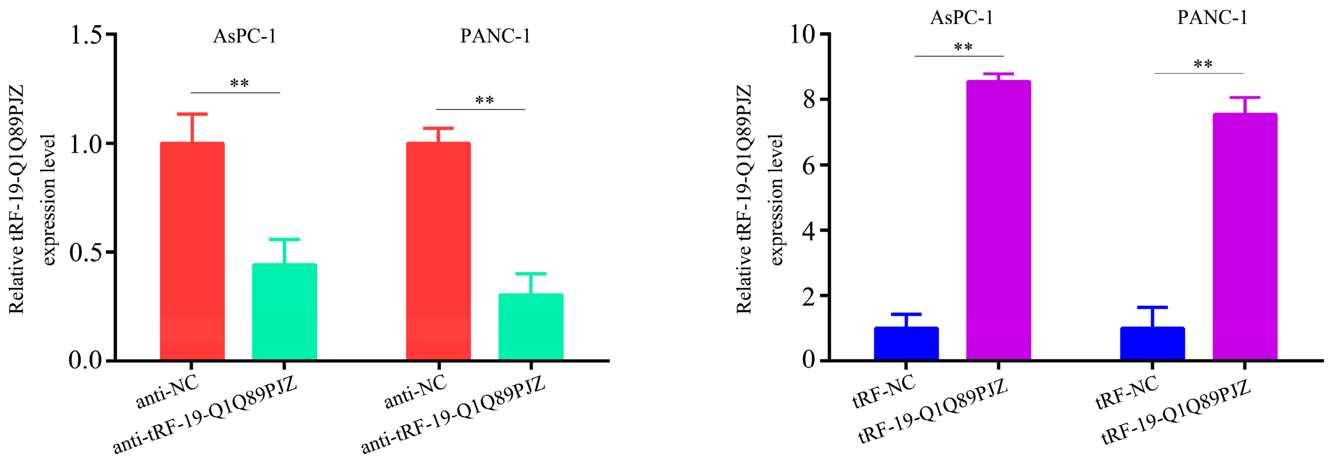

(**A**) (**B**)

Figure S2. tRF-19-Q1Q89PJZ inhibits the proliferation and metastasis of PC cells. (**A**,**B**) The transfection efficiencies of tRF-19-Q1Q89PJZ mimics and inhibitors in PC cells were verified by qRT-PCR. ** $p < 0.01$, $n = 3$. The control group was used for comparison. Data are shown as mean ± SD.

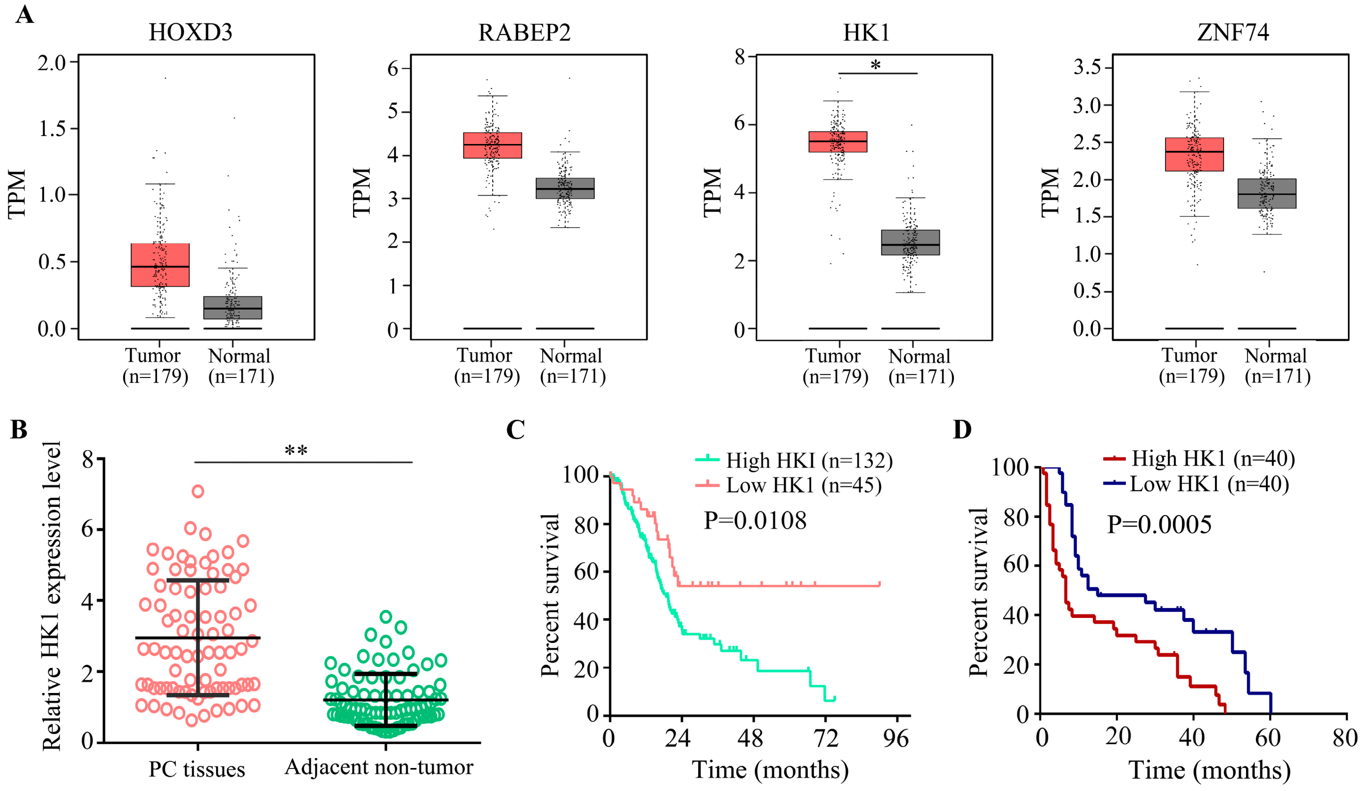

Figure S3. HK1 is a target of tRF-19-Q1Q89PJZ in PC. (**A**) HOXD3, RABEP2, HK1, and ZNF72 expression according to data from the TCGA database. (**B**) The expression level of HK1 was detected by qRT-PCR in 40 paired PC tissues and adjacent non-cancerous tissues. (**C**,**D**) KM plot showing overall survival of patients with low and high HK1 expression according to data from the TCGA database and our research cohort. * $p < 0.05$, ** $p < 0.01$, $n = 3$. The control group was used for comparison. Data are shown as mean ± SD.

Table S1. Correlation between pancreatic cancer characteristics and tRF-19-Q1Q89PJZ expression levels.

**tRF-19-Q1Q89PJZ Expression**					
**Features**	***n***	**Low**	**High**	**X²**	***p*-Value**
All cases	80	40	40		
Age				0.201	0.654
<60	42	22	20		
≥60	38	18	20		
Gender				0.052	0.82
Male	46	24	23		
Female	34	16	17		
Histological type				0.082	0.775
Adenocarcinoma	65	33	32		
Nonadenocarcinoma	15	7	8		
TNM stage				6.275	0.012
I and II	68	30	38		
III and IV	12	10	2		
Distant metastasis				6.347	0.012
Negative	71	31	39		
Positive	9	8	1		

Perineural invasion				7.5	0.006
Negative	48	18	30		
Positive	32	22	10		
Lymph node metastasis				8.455	0.004
No	41	14	27		
Yes	39	26	13		

Table S2. Different proteins overexpressing tRF-19-Q1Q89PJ and tRF-NC AsPC-1 based on the mRNA-Seq results.

ID	log2 (FC)	−log10 (*p*-Value)
HK1	−8.958096925	0.02188901
HOXD3	−7.683191273	9.61E-08
TRBV13	−7.278255115	2.28E-06
AKT1	−7.126616945	3.72E-06
TERT	−7.090244874	1.17E-16
MTOR	−7.019838839	0.001097199
FLG	−6.989382062	1.08E-05
KIF1A	−6.626080665	0.000187018
LCN15	−6.511264345	0.000209759
AC010327	−6.427303095	0.00051574
CU633967	−6.182664539	1.86E-10
LSP1	−6.176868862	0.012302053
FAM3B	−6.154749651	0.010767062
TMEM37	−5.962011864	0.016008065
IL4I1	−5.543216429	8.17E-05
HBE1	−5.538007987	3.02E-10
SUGCT	−5.511401153	4.31E-07
MYH16	−5.407388056	1.10E-15
PMF1-BGLAP	−5.027928222	3.01E-11
Z83844	−4.955049037	0.002213318
MAP1LC3C	−4.843532905	4.77E-31
LINC01224	−4.769397522	5.18E-05
HKDC1	−4.767715199	0.001218492
AC122697	−4.740271714	0.000663165
DOC2A	−4.399288515	1.82E-18
SEMA3D	−4.386602975	2.04E-14
AC026341	−4.378374333	4.85E-12
SNORA77B	−4.309811337	0.000845674
LCE1E	−4.110579818	0.000511758
SH3TC2-DT	−4.031775064	0.002666029
AL807752	−4.031659089	0.014598111
AL353572	−3.983601887	0.005389369
HAS2	−3.959597165	9.63E-18
UPK3BL1	−3.948983057	2.67E-08
HIF1A	−3.91647225	1.63E-14
AL138689	−3.853595197	2.00E-07
AC007383	−3.794431058	0.031504891
KRTAP2-3	−3.709032739	0.012610845
TRBV12-4	−3.636629404	2.12E-33
SNORA40	−3.630660299	0.021524851
ZNF672	−3.605899028	0.00012878

MX2	−3.598239255	4.45E-10
MYB	−3.580463932	2.55E-10
AL162253	−3.548999155	1.02E-15
RASGRP2	−3.502541123	0.025313843
SNORD88B	−3.481871741	0.000206838
SARDH	−3.466940974	0.000198121
LINC02310	−3.351849173	3.35E-08
AC068587	−3.341234121	5.80E-07
SH3TC2	−3.286535224	2.32E-13
TRBV12-3	−3.279494354	6.29E-13
STARD8	−3.278768994	3.96E-07
BNC2	−3.237343913	3.23E-15
ART5	−3.16012118	0.030428259
PLAU	−3.156096829	1.87E-37
AL359232	−3.155996174	0.003647358
AL031716	−3.138509114	0.047369293
SYN1	−3.131946455	0.010136089
CA9	−3.103962254	0.013701077
OAS1	−3.081954888	1.14E-09
WASH5P	−3.072797247	0.000843792
PRODH	−3.057823997	0.000991185
APLN	−3.036236402	1.01E-13
CENPS-CORT	−3.017845922	0.000756605
MIR155HG	−2.984182169	0.017687301
AC139100	−2.960332794	0.01060555
GAL	−2.944323975	3.44E-20
BSPRY	−2.943042032	0.006739302
BHLHA15	−2.918895167	6.35E-14
AL023803	−2.918228716	0.043459635
AC106845	−2.908487255	5.16E-07
SYS1-DBNDD2	−2.879207869	0.006961189
USP2-AS1	−2.848695217	0.00012138
RGS4	−2.846715155	1.07E-16
HPDL	−2.840637711	3.04E-12
ACSM3	−2.82569373	3.10E-09
AC023593	−2.812835638	4.17E-11
CYP4F3	−2.809985923	0.008856666
CASC19	−2.798914949	4.65E-08
INHBE	−2.787702179	4.10E-06
ADGRE2	−2.777557726	0.005906203
HAS2-AS1	−2.773383062	0.024705143
KCNH3	−2.753097032	0.006070469
ELFN1-AS1	−2.745736068	1.43E-08
LINC01358	−2.738827934	1.05E-20
FGD3	−2.733607821	9.53E-14
CALB2	−2.732021778	5.55E-09
SNORD17	−2.726989234	2.03E-06
SLC2A4	−2.724087552	3.49E-06
DDN	−2.712455456	2.48E-11
TMEM121	−2.712031478	3.50E-05
AL162258	−2.70721431	1.75E-12

LCE1F	−2.694510005	0.003066951
FNDC11	−2.690575922	0.001173517
OAS2	−2.680742555	5.20E-06
AC090673	−2.666784131	0.006957799
WDR4	−2.650750681	6.09E-16
POU5F1B	−2.627429793	0.012294116
NES	−2.613991341	1.94E-16
ZNF295-AS1	−2.611596204	9.47E-11
IPO4	−2.570576853	1.43E-15
PCAT1	−2.566090346	0.001740541
LINC01204	−2.56411091	0.002112839
AC010655	−2.555468783	0.009255842
AC079781	−2.528879779	0.02351109
DCST1	−2.524476881	0.043585921
PARD6G-AS1	−2.514586765	2.80E-07
ITGB3	−2.512992056	0.028596671
PALM3	−2.510283497	2.36E-09
SLC38A5	−2.503369886	1.10E-12
AL109917	−2.50269241	0.011920643
MCM2	−2.495856371	1.20E-09
MYOSLID	−2.495575874	4.07E-05
LRRN4	−2.478073934	2.54E-12
AC125611	−2.47754369	0.018666921
AC012313	−2.475281207	0.015682239
AC138904	−2.460791693	0.013370942
ACTBP11	−2.453476652	0.015981039
SLC19A1	−2.452297919	2.25E-12
TBX6	−2.448807856	3.35E-05
TJP3	−2.439072345	0.02377102
RCN3	−2.404272199	0.000249599
AC069148	−2.396884402	0.028587715
UCP2	−2.39247274	1.29E-10
LCN10	−2.389473076	5.69E-10
AL139156	−2.389139086	0.003947819
AC027307	−2.385698339	3.82E-05
CXCR6	−2.379912625	0.036970526
AC106820	−2.375654738	0.007614374
HGF	−2.357060361	1.46E-09
MCM3	−2.35629598	5.62E-19
SYTL1	−2.332651525	0.000208779
IFITM1	−2.3260823	6.10E-13
MT1L	−2.325141504	3.94E-11
MCM7	−2.319360425	6.65E-12
CKMT1B	−2.315432591	3.44E-11
TNFSF15	−2.314262472	9.64E-05
LINC01812	−2.293264283	8.88E-06
TLR3	−2.2922559	0.000185015
PLCB2	−2.288852479	7.07E-09
NKAIN1	−2.288559031	0.000124058
LARGE2	−2.262860094	1.63E-08
TTN	−2.256712228	5.37E-12

KRT8P14	−2.252043406	0.005301015
FAM110D	−2.245531079	0.036643858
ZNF664	−2.235941082	2.21E-05
POLE2	−2.234142217	1.46E-05
ZNF467	−2.220333227	1.30E-05
C1QTNF2	−2.218717112	0.038992792
AP002990	−2.212279268	0.037281295
AC005865	−2.212096689	0.001728277
FHAD1	−2.211651393	6.66E-06
CDH15	−2.208638409	7.62E-06
AC097263	−2.20584524	0.000365505
CACNA2D1	−2.203874132	0.000901476
SPATA12	−2.201369163	2.43E-05
ATXN2-AS	−2.200820775	0.038121549
AL359546	−2.198542187	0.001587506
TENT5B	−2.197998751	0.003766816
SNHG20	−2.18742796	4.76E-08
CA12	−2.180537708	9.85E-11
HMGA2-AS1	−2.165881212	0.000578554
RPSAP17	−2.154842647	0.003284491
AC112777	−2.154540565	6.06E-09
RAD54L	−2.15124983	5.45E-07
DIXDC1	−2.147839473	7.76E-06
HSD3B7	−2.143202203	6.89E-05
TFR2	−2.138709739	2.82E-07
RAD9B	−2.137903264	6.42E-05
IL17RE	−2.134086462	7.54E-09
MCM5	−2.133351218	2.74E-12
LMCD1	−2.127996648	1.01E-09
NRM	−2.127238346	2.66E-07
AC007191	−2.123705767	7.58E-10
PDZRN3	−2.122772993	2.28E-10
CLN6	−2.118429865	1.21E-14
AKR7A3	−2.103281125	8.92E-08
AC097658	−2.103102406	0.03858748
NAT16	−2.097342907	4.07E-05
AC087501	−2.093805838	6.83E-05
POLD1	−2.092663521	3.17E-11
AL138999	−2.092587228	0.000147087
LGALS3BP	−2.091710113	2.63E-07
ZBTB12	−2.088261505	1.40E-08
SLC25A10	−2.079837563	1.11E-07
FAM187A	−2.079699496	0.000171244
PAICS	−2.078141352	1.72E-12
NECTIN4	−2.071981187	0.004986447
NTNG2	−2.068923374	2.00E-10
LRRC45	−2.063806851	2.87E-07
LIG1	−2.061236508	1.66E-12
LINC02595	−2.051396643	0.037444616
AC097518	−2.049351827	0.000876275
DNAAF3	−2.046612352	1.25E-06

MRPS2	−2.044293005	8.06E-07
GAP43	−2.04033811	0.00565501
DERL3	−2.038787138	0.00017137
SORD2P	−2.038684443	4.55E-11
RPSAP52	−2.036560474	0.001035926
FAM81A	−2.030643514	2.08E-05
FAM222A-AS1	−2.030010395	6.50E-05
HELLS	−2.014000712	1.70E-08
TAFA3	−2.010353718	3.65E-05
CCDC74B	−2.004090203	0.000318703
AL359091	−2.001980275	0.003831656
MSN	−2.000599009	1.30E-07
ARRDC4	2.007013623	2.83E-08
MIR31HG	2.009185113	5.76E-05
TMEM269	2.010518201	0.009355446
AC138028	2.010632799	0.000938261
LIPH	2.011983019	7.28E-05
SEC31B	2.014142026	0.000542679
ZSWIM5	2.017395534	2.81E-11
RNF32-AS1	2.017846443	0.000322596
DCHS1	2.017873494	1.11E-05
AC007842	2.020896622	0.000186664
NOP14-AS1	2.02157997	6.87E-10
PARD3B	2.033826651	1.14E-14
AC104825	2.033868019	2.53E-05
MYOM1	2.041559475	0.000830724
CCN2	2.042471923	0.00736744
AL645608	2.043794247	0.000322212
AC141586	2.047433835	6.97E-09
RAB30	2.048332643	1.69E-10
ZNF425	2.067589701	2.56E-14
AC093525	2.073131226	8.68E-10
H2AC6	2.088562675	1.55E-12
COL6A3	2.097253824	0.025636161
IGIP	2.110466898	0.000147048
SPAG5-AS1	2.114962686	1.40E-07
AC024580	2.119279575	0.000117191
OVGP1	2.119937768	7.62E-06
AL139220	2.124480186	0.000123637
CCDC17	2.127212537	0.002905455
FGF7P6	2.131086771	2.09E-07
RN7SKP11	2.134476691	0.000651391
AC005280	2.158488841	0.005845398
TP53INP1	2.15947121	9.95E-15
LINC02086	2.162724702	4.61E-08
H2BC5	2.162932997	3.73E-11
SEMA3B	2.19194221	1.89E-05
AC020661	2.207451676	3.59E-06
COL5A2	2.210846116	0.000793329
AL139260	2.22186202	0.000102871
TUBB2B	2.233718435	0.000125008

MFAP2	2.235261735	2.13E-05
NT5E	2.245820842	1.57E-21
SIPA1L2	2.247772491	3.58E-07
PIP5K1B	2.255943495	0.000229417
KLF2	2.270297527	1.22E-16
TRPC1	2.285780347	3.14E-08
CFAP70	2.287858326	2.43E-05
ADAP2	2.296480984	0.000390838
ANKRD24	2.303118281	3.92E-07
ZCWPW2	2.30454887	1.89E-05
AC009108	2.30661888	4.78E-07
CDRT4	2.307045993	4.55E-05
YJEFN3	2.308324258	2.31E-06
PTGS2	2.311212289	1.42E-17
KCNH6	2.327463903	0.000144864
MIR22HG	2.338072506	5.06E-10
AC102953	2.36282791	2.84E-12
SMIM14	2.371461438	7.38E-17
EFR3B	2.374473534	7.33E-09
TRIM74	2.374537129	0.000966876
AC241952	2.385193936	6.21E-07
RPLP0P2	2.400240132	4.86E-11
CFAP53	2.400507187	9.75E-06
H2BC15	2.409945219	2.17E-09
AL596244	2.429054562	0.000115093
SELPLG	2.430346982	0.014954338
AC007728	2.45956857	3.06E-05
PCLO	2.472900851	4.62E-06
ASIC3	2.478298357	2.54E-08
GCNT4	2.482696292	1.24E-13
QRICH2	2.492305021	1.01E-11
EGR2	2.499469925	1.47E-18
RHOB	2.501782697	5.70E-13
MIR22	2.523183021	4.63E-07
SERPINI1	2.541671876	6.54E-09
AC005586	2.561510907	3.11E-08
AC046134	2.564275076	3.07E-07
SEMA3C	2.576359004	5.47E-07
ZFPM2-AS1	2.583450533	7.29E-06
AC087741	2.592489645	5.93E-07
H4C8	2.596645104	7.19E-19
SDCBP2	2.61575836	2.92E-18
ATXN1	2.621399949	9.47E-14
AL139385	2.641201943	7.45E-12
FCGBP	2.651995807	2.19E-05
FER1L4	2.664225923	1.28E-09
AL031118	2.674025399	0.000556428
FAM217B	2.758185134	1.25E-06
ISYNA1	2.760274971	1.18E-08
IL11RA	2.770885796	1.64E-05
KIF9-AS1	2.78375845	2.91E-12

ZNF540	2.790949177	8.01E-10
AOC2	2.798801826	8.20E-11
H2BC8	2.841707531	8.11E-13
AC016876	2.893655547	2.06E-14
NLGN3	2.921636581	2.83E-10
UCKL1-AS1	2.922571961	2.71E-07
NR4A3	2.935615251	8.00E-14
AC010323	3.016469344	0.032249446
FRY	3.017384481	3.66E-10
AC092143	3.032594316	1.09E-07
AOC3	3.037802726	3.51E-10
H2BC4	3.042201614	5.51E-16
ANKFN1	3.087341061	7.59E-08
HAPLN3	3.093064984	7.57E-05
CNTN4	3.094121039	4.08E-06
MZF1-AS1	3.100027872	1.27E-16
ZNF778	3.124074923	9.31E-30
KCND1	3.12425202	5.21E-11
REL	3.131550323	3.51E-11
UNC13D	3.256696539	1.30E-06
XYLT1	3.293966756	0.039184778
DNAH10OS	3.315308436	3.17E-07
SESN3	3.318639742	5.72E-11
AC118553	3.384831758	0.006399731
TMEM217	3.612588344	8.97E-14
LINC00513	3.740716311	1.47E-19
COL7A1	3.891975189	0.009476977
SLC9A3	4.181153523	0.001726205
PTEN	4.852118784	0.001068679
TNXB	4.980593166	2.81E-16

Table S3. The patients' basic clinical information.

Patient ID	Gender	Age (y)	Histological Type	TNM Stage	Lymph Node Metastasis	Distant Metastasis	Perinerual Invasion
1	Female	43	Adenocarcinoma	T1	N1	M0	Yes
2	Male	57	Adenocarcinoma	T3	N1	M1	Yes
3	Male	75	Adenocarcinoma	T1	N0	M0	Yes
4	Male	80	Adenocarcinoma	T1	N1	M0	Yes
5	Male	50	Adenocarcinoma	T1	N0	M0	Yes
6	Female	59	Adenocarcinoma	T2	N0	M0	Yes
7	Male	83	Nonadenocarcinoma	T3	N0	M0	Yes
8	Male	59	Adenocarcinoma	T2	N0	M0	Yes
9	Male	65	Adenocarcinoma	T1	N0	M0	Yes
10	Male	50	Nonadenocarcinoma	T3	N0	M0	Yes
11	Male	73	Adenocarcinoma	T1	N1	M0	Yes
12	Male	59	Adenocarcinoma	T1	N1	M0	Yes
13	Female	72	Adenocarcinoma	T3	N1	M1	Yes
14	Female	64	Adenocarcinoma	T2	N1	M1	Yes
15	Male	81	Adenocarcinoma	T2	N0	M0	Yes
16	Male	44	Nonadenocarcinoma	T3	N0	M0	Yes

17	Male	57	Nonadenocarcinoma	T2	N0	M0	Yes
18	Male	78	Adenocarcinoma	T1	N1	M0	Yes
19	Male	68	Adenocarcinoma	T1	N1	M0	Yes
20	Female	58	Nonadenocarcinoma	T2	N0	M0	Yes
21	Female	38	Nonadenocarcinoma	T2	N0	M0	Yes
22	Male	67	Adenocarcinoma	T1	N0	M0	Yes
23	Male	46	Adenocarcinoma	T1	N1	M0	Yes
24	Male	66	Adenocarcinoma	T1	N2	M0	Yes
25	Female	68	Adenocarcinoma	T1	N1	M0	Yes
26	Male	48	Adenocarcinoma	T3	N2	M1	Yes
27	Male	63	Adenocarcinoma	T1	N1	M0	Yes
28	Female	63	Nonadenocarcinoma	T3	N0	M1	Yes
29	Female	45	Adenocarcinoma	T1	N2	M0	Yes
30	Male	57	Adenocarcinoma	T2	N0	M0	Yes
31	Female	74	Adenocarcinoma	T1	N1	M0	Yes
32	Male	51	Adenocarcinoma	T2	N0	M0	Yes
33	Male	73	Nonadenocarcinoma	T3	N0	M0	Yes
34	Female	67	Nonadenocarcinoma	T3	N0	M0	Yes
35	Female	56	Adenocarcinoma	T3	N1	M1	Yes
36	Male	80	Adenocarcinoma	T1	N1	M0	Yes
37	Male	74	Adenocarcinoma	T1	N1	M0	Yes
38	Male	49	Adenocarcinoma	T1	N0	M0	Yes
39	Male	71	Adenocarcinoma	T1	N1	M0	Yes
40	Male	36	Adenocarcinoma	T1	N0	M0	NO
41	Male	59	Adenocarcinoma	T1	N2	M0	NO
42	Female	50	Adenocarcinoma	T2	N1	M1	NO
43	Male	59	Adenocarcinoma	T1	N0	M0	NO
44	Female	71	Adenocarcinoma	T1	N0	M0	NO
45	Female	55	Adenocarcinoma	T2	N0	M0	NO
46	Male	80	Adenocarcinoma	T1	N1	M0	NO
47	Female	68	Adenocarcinoma	T1	N1	M0	NO
48	Female	56	Adenocarcinoma	T1	N1	M0	NO
49	Female	72	Nonadenocarcinoma	T3	N0	M0	NO
50	Male	71	Adenocarcinoma	T1	N1	M0	NO
51	Male	57	Adenocarcinoma	T1	N0	M0	NO
52	Female	43	Adenocarcinoma	T1	N1	M0	NO
53	Female	34	Adenocarcinoma	T2	N0	M0	NO
54	Male	51	Adenocarcinoma	T2	N0	M0	NO
55	Male	67	Adenocarcinoma	T1	N1	M0	NO
56	Female	65	Adenocarcinoma	T1	N2	M0	NO
57	Female	77	Adenocarcinoma	T1	N0	M0	NO
58	Female	59	Adenocarcinoma	T1	N1	M0	NO
59	Male	57	Nonadenocarcinoma	T1	N0	M0	NO
60	Male	59	Adenocarcinoma	T1	N1	M0	NO
61	Male	55	Adenocarcinoma	T1	N1	M0	NO
62	Female	58	Adenocarcinoma	T2	N2	M1	NO
63	Female	72	Adenocarcinoma	T1	N1	M0	NO
64	Male	65	Nonadenocarcinoma	T1	N0	M0	NO
65	Female	48	Adenocarcinoma	T1	N1	M0	NO
66	Female	57	Adenocarcinoma	T1	N1	M0	NO
67	Male	56	Adenocarcinoma	T1	N2	M0	NO

68	Male	57	Adenocarcinoma	T2	N0	M0	NO
69	Female	62	Adenocarcinoma	T1	N1	M0	NO
70	Female	74	Adenocarcinoma	T1	N0	M0	NO
71	Male	41	Adenocarcinoma	T2	N0	M0	NO
72	Female	56	Adenocarcinoma	T2	N0	M0	NO
73	Male	59	Adenocarcinoma	T1	N0	M0	NO
74	Female	48	Adenocarcinoma	T1	N0	M0	NO
75	Male	59	Nonadenocarcinoma	T2	N0	M0	NO
76	Male	62	Adenocarcinoma	T2	N0	M0	NO
77	Female	62	Adenocarcinoma	T1	N1	M0	NO
78	Female	80	Nonadenocarcinoma	T2	N0	M0	NO
79	Female	85	Adenocarcinoma	T1	N0	M0	NO
80	Male	77	Nonadenocarcinoma	T2	N0	M0	NO

* T1: maximum tumor diameter (cm) ≤ 2; T2: maximum tumor diameter (cm) 2 to 4; T3: maximum tumor diameter (cm) ≥ 4; N0: no regional lymph node metastases; N1: metastasis in 1 to 3 regional lymph nodes; N2: metastasis in ≥4 regional lymph nodes; M0: Primary; M1: Metastasis.
